# DNA Methylation of Enhancer Elements in Myeloid Neoplasms: Think Outside the Promoters?

**DOI:** 10.3390/cancers11101424

**Published:** 2019-09-24

**Authors:** Raquel Ordoñez, Nicolás Martínez-Calle, Xabier Agirre, Felipe Prosper

**Affiliations:** 1 Área de Hemato-Oncología, Centro de Investigación Médica Aplicada, IDISNA, Universidad de Navarra, Avenida Pío XII-55, 31008 Pamplona, Spain; 2 Centro de Investigación Biomédica en Red de Cáncer (CIBERONC), 28029 Madrid, Spain; 3 Departamento de Hematología, Clínica Universidad de Navarra, Universidad de Navarra, Avenida Pío XII-36, 31008 Pamplona, Spain

**Keywords:** DNA methylation, Enhancer regions, myeloid neoplasms, acute myeloid leukemia (AML), myeloproliferative neoplasms

## Abstract

Gene regulation through DNA methylation is a well described phenomenon that has a prominent role in physiological and pathological cell-states. This epigenetic modification is usually grouped in regions denominated CpG islands, which frequently co-localize with gene promoters, silencing the transcription of those genes. Recent genome-wide DNA methylation studies have challenged this paradigm, demonstrating that DNA methylation of regulatory regions outside promoters is able to influence cell-type specific gene expression programs under physiologic or pathologic conditions. Coupling genome-wide DNA methylation assays with histone mark annotation has allowed for the identification of specific epigenomic changes that affect enhancer regulatory regions, revealing an additional layer of complexity to the epigenetic regulation of gene expression. In this review, we summarize the novel evidence for the molecular and biological regulation of DNA methylation in enhancer regions and the dynamism of these changes contributing to the fine-tuning of gene expression. We also analyze the contribution of enhancer DNA methylation on the expression of relevant genes in acute myeloid leukemia and chronic myeloproliferative neoplasms. The characterization of the aberrant enhancer DNA methylation provides not only a novel pathogenic mechanism for different tumors but also highlights novel potential therapeutic targets for myeloid derived neoplasms.

## 1. Enhancer Definition

Differentiation of the wide range of existing cell types requires the establishment of spatiotemporal patterns of gene expression during embryogenesis, but also during processes involving continuous differentiation through adulthood, such as hematopoietic differentiation [1]. Since their discovery in 1981 [2], enhancer elements have been demonstrated to play a key role in the regulation of transcriptional programs both under physiological and pathological conditions [3]. Enhancer regulatory elements function as integrated binding platforms for a variety of transcription factors [4], regulating the transcription of their target genes independently of orientation and at various distances from their target promoter [5]. The flexible nature of DNA allows enhancers to come into close spatial proximity to their target promoters through chromatin looping [6]. Remarkably, whereas promoter activation is largely invariant across cell types, enhancer regions have been demonstrated to be highly dynamic and correlate with cell-specific gene expression profiles [7,8,9,10]. Genome-wide studies have suggested that enhancers are likely to be the most dynamic elements in the genome, revealing more than 400,000 putative enhancer elements, pointing out to a key role in the spatiotemporal regulation of transcriptional programs [3].

**Figure 1:**
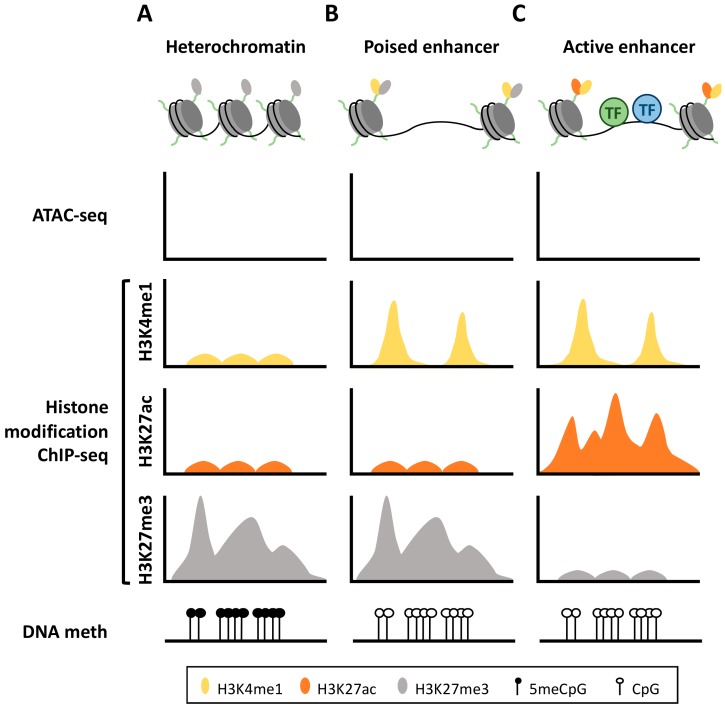
Chromatin landscape for heterochromatin, poised and active enhancer regions. (**A**) The inactive DNA is tightly packed around histone proteins marked with H3K27me3 modification, in the form of heterochromatin. This structure prevents any interactions of transcription factors (TF) with the DNA sequence. (**B**) When the enhancer region is pre-activated or poised, addition of H3K4me1 to the histone tails make the nucleosomes mobile, allowing their displacement to form highly accessible DNA regions, which get frequently demethylated. (**C**) Upon activation of enhancer region, nucleosomes flanking this region acquire H3K27ac, losing the repressing H3K27me3 mark, which subsequently recruits the corresponding transcription factors.

Until recently, the identification and functional annotation of enhancer elements had proved challenging, owing to the intrinsic dynamic nature of enhancers across cell types, their highly variable location, and the lack of a well-defined consensus sequence. The advances in epigenomic profiling technologies such as ChIP-seq (chromatin immunoprecipitation followed by high-throughput sequencing) have been effectively used to correctly annotate them, associating putative enhancer regions with the presence of monomethylation of lysine 4 in histone 3 (H3K4me1) and acetylation of lysine 27 in histone 3 (H3K27ac) (Figure 1). These two modifications, often in combination with chromatin accessibility data provided by DNase-seq (sequencing of DNase I hypersensitive sites) or ATAC-seq (assay for transposable-accessible chromatin-sequencing), provide a robust readout of genome-wide location of active enhancers, and have been utilized for enhancer annotation in a myriad of studies [8,11,12,13,14]. These chromatin marks are not simply passive modifications, for instance, in primed or poised enhancers associated with H3K4me1 modification, addition of the methyl group to the histone tail can prevent DNA methylation, facilitate nucleosome repositioning, and promote the binding of the so called “pioneer” factors responsible for enhancer activation [15,16]. Additionally, these marks can provide further functional information about the enhancer activation status, as presence of H3K27ac in adjacent nucleosomes distinguishes active enhancer states from those poised for activation, which are bivalently marked by H3K4me1 and H3K27me3 (trimethylation of histone 3 lysine 27) in specific cell types (Figure 1). Such poised enhancers have been defined to be at a “pre-activated” state, which allows rapid and temporal switch on/off, a feature of high relevance for complex differentiation programs, such as hematopoiesis [17].

## 2. Enhancer DNA Methylation

DNA methylation is a key mechanism for gene expression regulation. It consists in the addition of a methyl group (-CH_3_) to the 5-carbon position of cytosine bases in CpG dinucleotides by DNA methyltransferase enzymes (DNMTs), yielding 5-methyl-citosine (5mC). These enzymes are involved in both establishing de novo DNA methylation patterns (DNMT3A and DNMT3B) and their maintenance during cell division (DNMT1) [18]. Whereas DNA methylation mechanisms are well characterized, the DNA demethylation process is still controversial. On one hand, DNA methylation can be passively lost due to an inefficient maintenance during cell division. On the other hand, active DNA demethylation can occur by deamination of 5mC to thymine, catalyzed by Activation-induced Cytidine Deaminase (AID) enzyme; or by hydroxylation to 5-hydroxymethylcytosine (5hmC), catalyzed by the Ten-Eleven Translocation protein family (TET1, TET2 and TET3) [19]. Some recent studies have revealed preferential activity of TET protein family on enhancer regions during embryonic or other physiological processes such as Forkhead Box P3 (FOXP3) expression in T-lymphocytes [20,21,22,23] or the DNA demethylation of super-enhancer activity of AID by TET proteins during the B cell differentiation [24].

Cytosine methylation to 5mC involving CpG dinucleotides has been predominantly implicated in transcriptional silencing, particularly when located in promoter regions. Remarkably, DNA methylation can also take place outside promoters (i.e. gene bodies or intergenic regions) [25]. Although the mechanisms are not fully characterized, non-promoter DNA methylation has also been demonstrated to control gene expression through regulation of transcriptional elongation [26], determination of alternative promoters [27], regulation of mRNA splicing [28] or by interfering with binding of transcription factors to enhancer regions [29,30,31]. Such DNA methylation outside promoter elements has been shown to be more dynamic and more tissue-specific than canonical promoter methylation, largely overlapping with enhancer functionality [25,32,33]. In fact, inactive enhancers display higher levels of DNA methylation, whereas hypomethylation of enhancer DNA is associated with transcription factor binding and subsequent transcriptional activation [34,35] (Figure 1). However, it is worth noting that epigenetic regulation of enhancer activation does not rely only on DNA methylation, as histone modifications cooperate with DNA methylation to control accessibility of chromatin to key transcription factors in a cell-specific and time-dependent manner [36]. Epigenetic-mediated enhancer activation/inactivation has been observed throughout embryonic development [37], influencing, for example, primordial embryonic stem cells differentiation or neural-glial specification [38,39]. Enhancer DNA methylation also influences terminal differentiation processes in mature individuals, such as T-cell lineage specification or granulopoiesis [29,40]. Accordingly, deregulation of DNA enhancer methylation translates into pathological states, such as neoplastic transformation, where aberrant enhancer methylation contributes to the malignant phenotype inducing cellular de-differentiation in both solid and hematological tumor cells [41,42,43,44,45].

Although the defining features of enhancer regions are common (high levels of H3K4me1 and H3K27ac histone marks as well as DNA hypomethylation), their cell-type specific function should be determined by the binding of additional factors that may further alter the chromatin structure or influence transcription. Moreover, these epigenetic modifications are deposited in a cell-type dependent context by distinct histone chaperones or chromatin modifying enzymes, subsequently recruited to the enhancer regions by sequence-specific DNA binding proteins or other factors [46]. The coordinated recruitment of multiple transcription factors and chromatin modifiers to enhancer regions involves complex regulatory machinery, which will be summarized in the following section.

## 3. Epigenetic Machinery Associated with Enhancer Regulation

Most genome-wide studies show and inverse correlation between histone H3K4 methylation and DNA methylation, in putative enhancer regions. The interaction between both epigenetic modifications is regulated by a cross-talk among: (1) histone methyltransferases (HMTs), which can recognize hypomethylated DNA through methyl binding domains (MBDs) and zinc finger CXXC domains, and (2) DNMTs, which contain domains recognizing methylated histones [15]. Six different lysine-specific HMT have been shown to catalyze H3K4 methylation in mammal cells: four Mixed Lineage Leukemia enzymes (MLL1-4) and two SET domain containing proteins (SET1A and SET1B). Specifically, MLL3 and MLL4 are recognized examples of enzymes responsible for organizing genome-wide H3K4me1 levels at enhancer elements [47]; this event frequently occurs upon DNA demethylation [48,49], rendering enhancer structures accessible for activation. MLL proteins have been reported to interact with cell-type specific and signaling-dependent transcription factors [38,50,51], suggesting that enhancer activation can be orchestrated by specific transcription factors. Transcription factor binding can directly activate gene transcription of enhancer regions; however, this event requires recruitment of different co-activator proteins. CREB binding protein (CREBBP or CBP) and p300 are two examples of ubiquitously expressed histone acetyltransferases (HATs) constituting a co-activation complex that targets enhancer regions [52]. In fact, p300/CBP complexes have been successfully used for genome-wide enhancer mapping in different cell types and tissues [7,53,54]. An additional layer of complexity comes from the bivalent state of enhancers, which is marked by the presence of acetylated residues in neighbor histones, such as H3K27ac [14,17,55] (Figure 1). It remains to be demonstrated if the acetylation is directly responsible for the transition from poised to active enhancer, or on the contrary is only a passive marker of enhancer activation.

Deposition of enhancer-related histone marks is closely co-regulated with enhancer DNA methylation. However, the hierarchy of these enhancer epigenetic modifications remains unclear. On the one hand, there is evidence of regulation of DNA methylation through specific histone mark deposition, as demonstrated by the recruitment DNMTs to sites of unmethylated histones (H3K4me0) and the activity of chromatin-interacting complexes, such as the ATRX-DNMT3-DNMT3L [56,57]. This later complex specifically recognizes H3K4 methylation and guides DNA methylation activity of DNMT3A towards enhancer elements [57,58,59]. In contrast, some recent studies define DNA methylation as the leading epigenetic modification, instructing histone mark deposition through recruitment of methyl-CpG binding proteins (MBD) [60] and exclusion of the PRC2 complex from demethylated enhancers and promoters [61]. As an example, mouse embryonic stem cells devoid of DNA methylation by *DNMT3A* knockout, show H3K4me3 and H3K27ac chromatin marks as the fundamental modifications regulating gene transcription. However, these histone marks were reversed upon reconstitution of *DNMT3A* expression resulting in downregulation of gene expression. Therefore, although it is plausible that the regulation between DNA methylation and histone mark deposition would also be cell-type specific, further research is required to expand our knowledge of the chromatin/DNA methylation co-regulation, specifically in hematopoiesis [62].

## 4. Enhancer DNA Methylation in Myeloid Diseases

**Figure 2:**
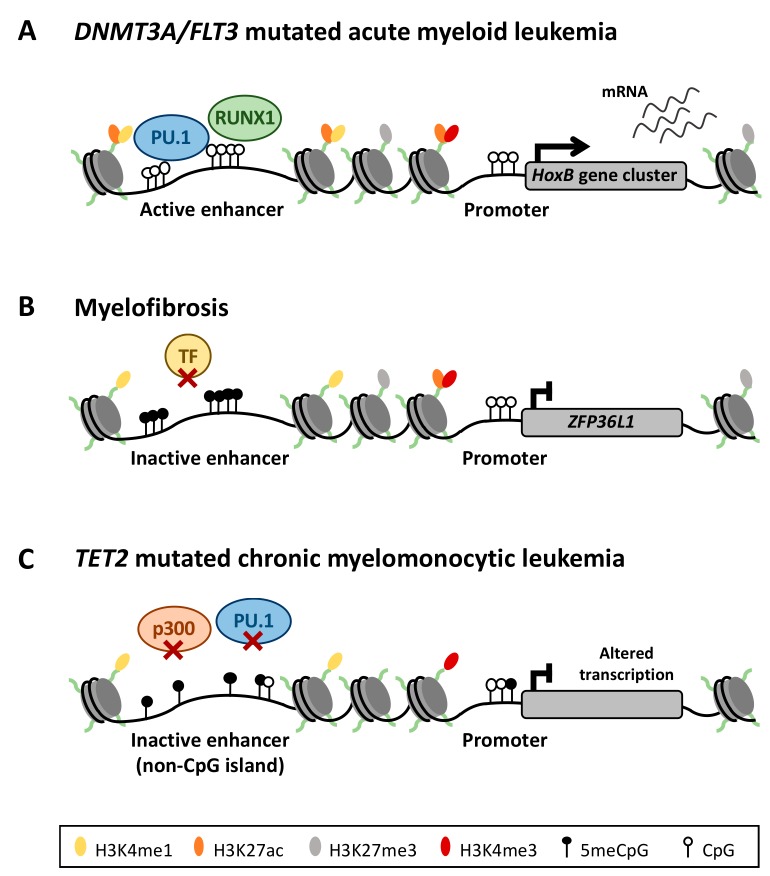
Aberrant DNA methylation of enhancer regions deregulates the transcriptional program of myeloid neoplasms. (**A**) In *DNTM3A/FLT3* mutated AML, DNA demethylation activates new and poised enhancers, making accessible binding sites for transcription factors implicated in myeloid differentiation, such as RUNX family transcription factor 1 (RUNX1) or Spi-1 proto-oncogene (SPI1 or PU.1). Such aberrant regulatory landscape induces a leukemic transcriptome, altering for example the expression of the *HOXB* gene cluster. (**B**) DNA methylome of myelofibrosis patients is characterized by an aberrant enhancer DNA methylation signature, which alters the gene expression pattern of relevant genes for neoplastic transformation, such as tumor-suppressor gene *ZFP36L1*, silenced in patients after aberrant DNA enhancer hypermethylation. (**C**) *TET2* mutated chronic myelomonocytic leukemia (CMML) cells shows an aberrant methylated DNA landscape, overlapping with regulatory enhancer regions in normal cells. Such DNA hypermethylation prevents binding of key regulators for myeloid differentiation, such as p300 or PU.1, altering the transcriptional program of these cells.

Hematopoiesis is a well-defined differentiation process that involves widespread chromatin remodeling. Recent studies demonstrate that the establishment, activation or decommission of enhancer regions through different lineage commitment steps is crucial for proper cell differentiation [9]. A clear example of this phenomenon occurs in normal granulopoiesis, where enhancers seem to suffer an increase of DNA methylation in the initial stages of differentiation (from the common myeloid progenitor to granulocyte-monocyte progenitor), followed by the loss of enhancer DNA methylation in mature granulocytes, which correlates with gene expression patterns in these cells [32,55]. Monocyte differentiation is also dependent on the expression of a specific enhancers repertoire, tightly regulated by epigenetics [63,64]. Evidence of such a prominent role of enhancer DNA methylation in hematopoiesis leads us to assume that the aberrant DNA methylation of cancer cells can potentially affect enhancer regions, thereby deregulating the cell transcriptome in hematological neoplasms (Figure 2). 

### 4.1. Aberrant Enhancer DNA Methylation in Acute Myeloid Leukemia

Acute myeloid leukemia (AML) is a hematologic neoplasm characterized by an impaired differentiation process, leading to an accumulation of immature blasts in the blood [65]. Recent studies have demonstrated that AML clones feature abnormal DNA methylation preferentially in CpG sites mapped to enhancer regions, with a striking predominance of hypomethylation [66]. AML with specific cytogenetic and mutational profiles shows differential DNA methylation profiles, in particular, *DNMT3A* and *IDH* gene mutations have been shown to have antagonistic patterns of enhancer DNA methylation, suggesting that epigenetic consequences of these mutations could be largely contributing to their malignant phenotype. Interestingly, AML with *CEBPA* silencing represents an exception to this, featuring hypermethylation of promoter regions and little changes in enhancer DNA methylation, in line with the distinct clinical and biological characteristic of this AML subtype [66].

Additional studies published by Qu Y et al, have shown that not only AML patients with gene mutations but also unmutated AML harbor an aberrant DNA methylome compared to healthy CD34+ cells, which is significantly altered at enhancer regulatory regions [67]. Genome wide profiling of these cells have linked such changes in DNA methylation to chromatin mark deposition in enhancer regions, showing significant correlation between DNA hypomethylation and active chromatin marks (DNAse sensitivity, H3K4me1, H3K4me3 and H3K27ac). Consequently, DNA demethylation activates new and poised enhancers in AML, causing a leukemia-associated transcriptome in these cells [67] (Figure 2A). Importantly, aberrant enhancer DNA methylation in AML has been shown to be independent of the expected differentiation-induced changes at these sites, suggesting that this aberrant DNA methylation profile is unique to the pathological state in AML and could be a central event in leukemogenesis [68]. Moreover, DNA methylation levels at specific enhancer regulatory regions could be used to predict overall survival of AML patients [68].

The studies conducted by Yang L et al, provide further evidence supporting the key role of enhancer DNA methylation in AML development and its association to DNMT3A activity, known to be frequently altered in myeloid neoplasms and particularly in AML [69,70,71]. Their experiments with *DNMT3A* knockout mice demonstrate that loss of DNA methylation, in the context of a heterozygous *DNMT3A* knockout, coupled with *FLT3-ITD* mutation are capable of developing *de novo* AML in affected mice [72]. Furthermore, *DNMT3A* knockdown was associated with predominant changes in DNA methylation at enhancer sites, whose functional analysis revealed potential binding sites for many of the transcription factors known to drive myeloid differentiation (e.g., *RUNX1*, *PU.1*) [72] (Figure 2A). These observations have also been confirmed in patient samples with *DNMT3A* R882 mutation, which displayed enrichment of DNA hypomethylation at enhancer sites, similar to those observed in the *DNMT3A* knockout model. Gene expression pathway analysis of the gene signatures related to these hypomethylated enhancers also revealed enrichment of functions related to hematopoietic development, among which *HOXB* gene cluster was prominent [72] (Figure 2A). This gene cluster has been previously recognized as an important player of hematopoiesis [73] and shows significant overexpression in AML patients [74]. 

Further evidence pointing towards the importance of enhancer regulatory regions in AML pathogenesis comes from a phenomenon called enhancer hijacking [75], which consists of recurrent translocations involving enhancer elements in the myeloid compartment. AML patients with inv(3) or t(3;3) are characterized by repositioning of *GATA2* enhancer into the *EVI1* locus. This result in a double effect of inappropriate *EVI1* upregulation coupled with downregulation of *GATA2*, proven drivers of AML development [76,77]. 

Overall, it is now accepted that enhancer deregulation is a frequent and predominant alteration of AML cells. The consequences of these alterations still remain to be fully investigated, however, it seems clear that enhancer DNA methylation couples with chromatin mark deposition to govern enhancer functionality, ultimately affecting transcription factor binding to the enhancer regions and shaping the transcriptomic profile of AML cells.

### 4.2. Deregulation of the DNA Methylation Signature in Philadelphia Chromosome-Negative Myeloproliferative Neoplasms

Philadelphia chromosome-negative myeloproliferative neoplasms (MPN), including polycythemia vera (PV), essential thrombocythemia (ET) and primary myelofibrosis (MF), are characterized by a clonal transformation of hematopoietic progenitors leading to expansion of fully differentiated myeloid cells [78]. Primary MF carries the worst prognosis of all MPN, in which recent reports have associated the phenotypic characteristics of MF patients with an aberrant DNA methylation profile [79]. Initial studies focused on promoter DNA methylation identified a limited number of differentially methylated CpG sites in MPN when compared to healthy donors, being unable to find specific DNA methylation profiles for each different malignancy (i.e., MF, PV or, ET) [80]. The findings on promoter DNA methylation indirectly indicate that aberrant DNA methylation could be targeting CpG sites outside of the canonical promoter region, as it has been later demonstrated. Work by Martinez-Calle et al, has indeed shown that the DNA methylome in MF is characterized by a pathological enhancer DNA methylation signature, independent of *JAK2V617F* mutation status. This aberrant enhancer DNA methylation correlated with changes in expression of relevant genes for hematopoietic differentiation revealing a gene expression profile that is likely to contribute to the malignant phenotype [43]. One representative example of this phenomenon is the silencing of *ZFP36L1* transcriptional regulator mediated by DNA hypermethylation of its associated enhancer in MF patients (Figure 2B). This gene behaves as a tumor suppressor gene in MF, highlighting the crucial role of aberrant enhancer DNA methylation in deregulating the expression of key genes for neoplastic transformation. Additional genome-wide studies have also confirmed differential DNA methylation profile of MF samples, showing an enrichment of differentially methylated CpGs in regions marked by the enhancer-related H3K4me1 histone mark [79].

Overall, DNA methylation landscape of chronic MPN is significantly altered, deregulating its transcriptional profile and affecting a significant number of relevant signaling pathways. More importantly, the enhancer signature of these patients is largely affected by changes in DNA methylation patterns, as demonstrated also for AML cells, suggesting that enhancer DNA methylation is indeed common to myeloid malignant transformation in both cases. The specific role of enhancer DNA methylation in the early transforming events and the maintenance of the malignant phenotype continues to be actively investigated.

### 4.3. DNA Methylation in TET2 Mutated Chronic Myelomonocytic Leukemia

Enhancer DNA methylation is also relevant for other myeloid neoplasms such as chronic myelomonocytic leukemia (CMML). This rare clonal hematological disorder is characterized by the aberrant transformation of the hematopoietic stem cell compartment, displaying overlapping features of myelodysplastic syndromes (due to defective hematopoiesis), and myeloproliferative neoplasms (due to aberrant hyperactivated hematopoiesis) [81]. Pérez et al demonstrated that changes in DNA methylation in CMML patients seem to be associated with *TET2* mutations [82]. TET2 is an epigenetic regulator that has been shown to catalyze the conversion of 5mC to 5-hydroxymethyl-cytosine (5hmC), leading to active DNA demethylation of the modified CpG sites. It plays important roles in normal hematopoiesis, including stem cell self-renewal, lineage commitment and terminal differentiation of monocytes [83,84,85]. *TET2* has been recognized as a tumor-suppressor gene, which is frequently mutated in human hematopoietic malignancies. *TET2* mutations can lead to frame-shift, new stop-codons, in-frame deletions, or highly conserved amino acid substitutions [85]. Such mutations have been demonstrated to impair TET2 catalytic activity, resulting in reduced 5hmC levels in affected cells. Interestingly, such aberrant DNA methylation profile detected in *TET2*-mutated CMML patient samples was significantly enriched outside CpG islands, which overlap with enhancer regulatory regions enriched for PU.1 transcription factor and p300 regulatory complex (Figure 2C), as was further validated in different studies [86,87,88]. However, these studies have also revealed a heterogeneous behavior of 5mC and 5hmC profiles in CMML patients, suggesting that epigenetic changes in this neoplasm are driven by additional mechanisms beyond the inactivation of TET2 protein.

Homozygous and heterozygous mutations in *TET2* gene are recurrent in hematopoietic malignancies besides CMML (frequency ranging from 30 to 60%), including myelodysplastic syndromes (20–35%), AML (12–34%) or lymphoid malignancies (2–33%) [85]. Moreover, *TET2* deletion has been demonstrated to be sufficient to cause both myeloid and lymphoid malignancies in mice [89]. Biological consequences of *TET2* mutations are thought to extend beyond DNA demethylation, as *TET2* might also participate in the regulation of the immune system, processes of the DNA repair response and may even cooperate with other gene mutations to promote neoplastic transformation [85]. Additional studies are required to shed light on the implications of *TET2* mutations in regulation of the DNA methylation landscape of normal and malignant hematopoietic cells.

## 5. Diagnostic and Therapeutic Implications

In spite of the quickly accumulating evidence of enhancer-specific DNA methylation changes and its potential relevance for myeloid malignancies, the therapeutic restoration of physiological DNA methylation status remains utopic, although some advancements have been made. Hypomethylating agents (i.e., *DNMT3A* inhibitors) are a clinical reality in the treatment of myelodysplastic syndromes and AML [90,91] providing modest but meaningful improvement in response rates and survival of patients. However, linking the DNA hypomethylating effect to efficacy of these agents has remained elusive, in fact, the precise mechanism of action responsible for the control of leukemic clones is still largely unknown. These agents have a broad specificity for DNA methylated sites of the genome and many off-target effects. Therefore, this is unlikely to represent a solution for the precise and temporary manipulation of DNA methylation that is required for a targeted epigenetic therapy.

The Bromodomain and Extra-Terminal Domain (BET) proteins constitute a family of epigenetic readers that can recognize acetylated lysine residues in histones, recruiting specific effector proteins to active chromatin regions, such as promoters and specially enhancers of active genes. BET proteins have been reported to play a crucial role in regulating gene transcription during cell proliferation and cell differentiation [92], such as the mechanistic studies conducted by Dey et al, revealing that BET protein BRD4 binds preferentially to super-enhancer structures contributing to the expression of lineage specific genes in the myeloid compartment [93]. Besides their role in physiological cellular processes, BET proteins have been also identified as key players in the maintenance of the neoplastic phenotype [94]. Indeed, several lines of evidence point towards targeting BET proteins as a new strategy for cancer treatment. For these reasons, BET protein inhibitors (BETi) are a novel class of epigenetic drugs that have experienced an exponential development over the last decade. The first published clinical results of a BETi resulted in cell growth inhibition, cell-cycle arrest and apoptosis of AML cell lines, driven by the decreased expression of *BRD2* and *BRD4* BET genes and relevant oncogenes, such as *c-MYC* [95,96] or NF-kappa β complex genes [97,98]. Mivebresib and OTX015 are examples of BETi tested in AML patients [97,98], showing an acceptable safety profile. BETi in fact constitute the first class of enhancer-directed epigenetic therapy reaching clinical development and have the potential to become part of the therapeutic armamentarium for AML and other hematological malignancies, such as B-cell lymphomas [87,88]. 

The main obstacle for epigenetic therapy is the unselective activity of DNMT inhibitors and BETi, potentially affecting the expression of genes that could result in unpredictable biologic consequences. To overcome these obstacles several alternatives have been explored, including TALEN or Zinc-finger proteins coupled with TET2 demethylase [99] as well as methyltransferase domains [100]. Preliminary experiments with these engineered proteins have successfully altered the DNA methylation status and the expression of a single locus [101]. These techniques hold promise for a tailored epigenetic therapy that can be applied to the diseased hematologic precursors; however, they remain in their infancy.

Enhancer DNA methylation can also serve as a biomarker for treatment response. Genome-wide DNA methylation studies in CMML have revealed a signature of differentially DNA methylated regions that associate with responders to DNA hypomethylating agent Decitabine [102], that is now widely used in clinical practice for myelodysplastic syndromes and AML. The enhancer DNA methylation profile associated with response to Decitabine leads to downregulation of specific cytokines in CMML patients; indeed, exogenous administration of these cytokines to responders reduced Decitabine efficacy. This implies that the enhancer DNA methylome may constitute a promising biomarker to predict response to therapeutic agents. Moreover, such a resistant phenotype could be at least partially and temporarily reversed to enhance therapeutic response [103].

Finally, apart from therapeutic manipulation of enhancer DNA methylation, this epigenetic mark could also be envisioned as diagnostic tool. Some epigenetic programs are sufficiently conserved in cancer cells, as has been widely demonstrated in genome-wide studies [104,105], allowing for early detection of tumoral cells based on DNA methylation abnormalities in routine clinical samples. This is already a reality for colorectal and breast cancer: the identification of vimentin gene DNA methylation [106] in blood samples and the *PTIX2* gene in breast cancer are two representative examples [32]. In AML, *CEBPA* gene DNA methylation has also been proposed as a favorable prognostic biomarker at diagnosis [107,108]. None of these biomarkers are specific for enhancer regions, but given the predominant role of enhancers in cancer-specific gene expression, identification of key aberrantly DNA methylated enhancers in tumoral samples can potentially turn them into useful clinical biomarkers.

## 6. Conclusions

Deranged enhancer DNA methylation is emerging as a prominent feature of myeloid neoplasms, adding an additional layer of complexity to the already entangled epigenetic landscape of these diseases. As has been described above, enhancer regulation is also crucial for hematopoietic and myeloid differentiation; hence, it is of no surprise that they also play an important role in neoplastic transformation. Much remains to be learnt from the dynamic and complex regulation of the DNA methylation status of enhancer regions before it can be translated into the diagnostic and therapeutic fields. Enhancer regulation is certainly a nascent and promising topic in epigenetic research that is expected to yield significant advancements in the next decades.
