# Correction: Ordoñez, et al.; DNA Methylation of Enhancer Elements in Myeloid Neoplasms: Think Outside the Promoters? *Cancers* 2019, *11*, 1424

**DOI:** 10.3390/cancers12071885

**Published:** 2020-07-13

**Authors:** Raquel Ordoñez, Nicolás Martínez-Calle, Xabier Agirre, Felipe Prosper

**Affiliations:** 1 Área de Hemato-Oncología, Centro de Investigación Médica Aplicada, IDISNA, Universidad de Navarra, Avenida Pío XII-55, 31008 Pamplona, Spain; rordonez.1@alumni.unav.es (R.O.); nmartinezc@unav.es (N.M.-C.); 2 Centro de Investigación Biomédica en Red de Cáncer (CIBERONC), 28029 Madrid, Spain; 3 Departamento de Hematología, Clínica Universidad de Navarra, Universidad de Navarra, Avenida Pío XII-36, 31008 Pamplona, Spain

The authors would like to make a correction to their published paper [1].

In Figure 1, the ATAC-seq section appears empty, and should be changed for a complete Figure 1. The correct Figure 1 is as below:

The change does not affect the review results.

The rest of the manuscript does not need to be changed. The authors would like to apologize for any inconvenience caused. The manuscript will be updated, and the original will remain available on the article webpage.

## Figures and Tables

**Figure 1:**
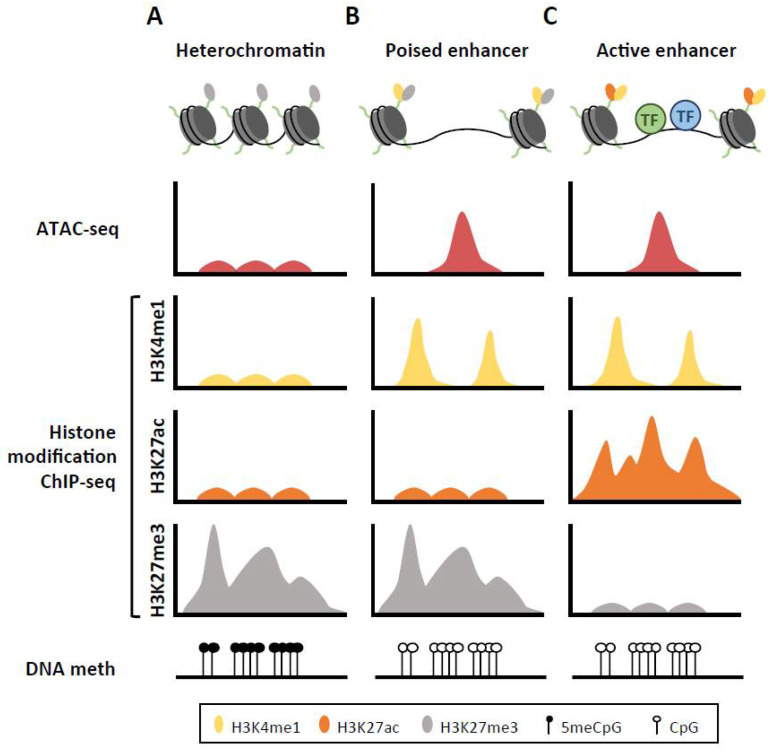
Chromatin landscape for heterochromatin, poised and active enhancer regions. (**A**) The inactive DNA is tightly packed around histone proteins marked with H3K27me3 modification, in the form of heterochromatin. This structure prevents any interactions between transcription factors (TF) and the DNA sequence. (**B**) When the enhancer region is preactivated or poised, addition of H3K4me1 to the histone tails make the nucleosomes mobile, allowing for their displacement to form highly accessible DNA regions, which get frequently demethylated. (**C**) Upon activation of enhancer region, nucleosomes flanking this region acquire H3K27ac, losing the repressing H3K27me3 mark, which subsequently recruits the corresponding transcription factors.
